# Brachial pulse waveform characteristics predict development of organ failure in septic patients

**DOI:** 10.1186/cc12136

**Published:** 2013-03-19

**Authors:** S Kazune, I Jagmane

**Affiliations:** 1 Hospital of Traumatology and Orthopaedics, Riga, Latvia

## Introduction

Pulse waveform characteristics (pulse wave transit time and augmentation index) are measures of arterial stiffness. Previous studies have found an association between severity of acute inflammatory states and increased arterial stiffness but it is not known whether non-invasive pulse waveform analysis could predict development of multiple organ failure in septic patients. The purpose of this study was to evaluate the photoplethysmographic brachial artery pulse wave transit time and augmentation index and their changes in response to induced forearm ischemia in septic ICU patients and correlate these indices to the development of subsequent end organ damage.

## Methods

A prospective observational study in patients with sepsis within 24 hours of admission. Severity of sepsis was assessed with APACHE II score (median 18.5) and SOFA score (median 7.5). Three-minute signal recording was done concurrently from the brachial artery at the elbow and the radial artery at the wrist with an originally designed photoplethysmograph at rest and after 5 minutes of induced forearm ischemia. Recordings were analyzed to obtain the pulse wave transit time and augmentation index at rest and 60 seconds after induced ischemia. The SOFA score was recalculated at 48 hours post recording.

## Results

We studied 14 consecutive general ICU patients. There was a negative linear relationship between the pulse wave transit time (median 22.6 ms) at rest and increase in SOFA score in 48 hours (*P *= 0.02, *r = *0.96). The postischemic pulse wave transit time increased in all patients (median 25.7 ms) but no association was found between the proportion of increase and subsequent change in SOFA. Correlation between rest (median 7.6) and postischemic (median 7.2) augmentation index and 48-hour SOFA scores was not statistically significant (*r *= 0.57, *P *= 0.46).

## Conclusion

This study indicates that in early sepsis pulse waveform characteristics could predict the risk of developing end organ failure. The pulse wave transit time is more robust than the augmentation index and could be easier to use in patients with poor perfusion. Vascular reactivity indices do not seem to have predictive value in this context.
